# A novel cystatin in *Psoroptes ovis* var. *cuniculi*: molecular characterization, serodiagnostic potential, and its anti-inflammatory property on rabbit peripheral blood mononuclear cells

**DOI:** 10.1186/s13071-024-06483-3

**Published:** 2024-09-19

**Authors:** Xiaobin Gu, Fusheng Yang, Ce Wang, Jing Xu, Yane Li, Youping Liang, Je Fan, Fangyan Wu, Ran He, Hui Wang, Yue Xie

**Affiliations:** Department of Parasitology, College of Veterinary Medicine, Sichuan Agricultural University, Chengdu, Sichuan People’s Republic of China

**Keywords:** *Psoroptes ovis* var. *cuniculi*, Cystatin, Inhibition activity, Serodiagnosis, Antiinflammatory property

## Abstract

**Background:**

The ectoparasite *Psoroptes ovis* var. *cuniculi* causes substantial economic losses to the global rabbit industry. Currently, microscopy for identifying *Psoroptes* mite in skin scrapings, as the “diagnosis gold standard,” remains a challenge owing to its poor sensitivity in detecting low-level and/or early stage mite infestations. Additionally, *Psoroptes* infestations rapidly trigger cutaneous inflammation, thus the mites might produce some molecules to deal with the harmful effects of inflammation for their long-time survival on the host skin, but these molecules are still mostly unknown.

**Methods:**

To seek a sensitive diagnostic method and illuminate the new antiinflammatory molecules, we characterized a novel cystatin of *P. ovis* var. *cuniculi* (PsoCys) using bioinformatics and molecular biology methods.

**Results:**

The results showed that PsoCys comprised the classical features of the type II cystatin superfamily including an N-terminal glycine residue, a central QXVXG motif, and a C-terminal LW motif. In mixed stages of mites, the transcription level of PsoCys was significantly higher in “fed” mites than in “starved” mites (*P* < 0.001), and among the different life-cycle stages of “fed” mites, the expression of PsoCys was higher in adult males than in larva, nymph, and adult females (*P* < 0.001). The established indirect ELISA based on recombinant PsoCys (rPsoCys-iELISA) presented 95.4% sensitivity and 95.7% specificity. The area under the receiver operating characteristic curve (AUC) for this method was 0.991, indicating its excellent diagnostic performance. Moreover, rPsoCys-iELISA had advantages over microscopy for detecting low-level and/or early stage mite infestations (90% versus 40% in artificial infestation cases at 3 weeks post-infestation; 61.9% versus 22.6% in clinical cases). In addition, rPsoCys could inhibit the activity of papain and cathepsin B in vitro, and significantly suppressed mRNA levels of toll-like receptors (TLR 1, 2, 4, and 6) and downstream molecules (NF-κB, p38, MyD88, IL-10, and IFN-γ) in LPS-stimulated rabbit PBMCs, indicating its anti-inflammatory property.

**Conclusions:**

Our findings indicated that PsoCys was a novel type II cystatin of *Psoroptes* mites, and it served as a potential serological diagnostic antigen for detecting low-level and/or early stage mite infestations, as well as a novel anti-inflammatory molecule of *Psoroptes* mites.

**Graphical abstract:**

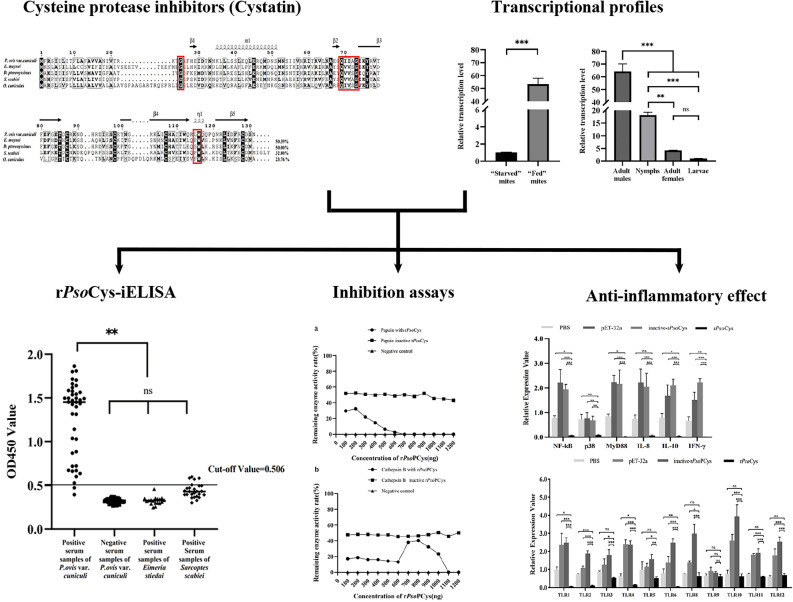

## Background

*Psoroptes ovis* var. *cuniculi* is a widespread ectoparasite in the commercial rabbit industry worldwide, with high contagiousness and over 70% incidence rate in rabbit industry. Infestation with *Psoroptes* mites cause pruritus, scab formation, weight loss, and even death, resulting in significant economic losses for the global commercial rabbit industry [1–3]. Currently, microscopic detection of *Psoroptes* mite in skin scrapings is deemed to be the “gold standard” for diagnosis. Therefore, only cases with visible scabs can be used for microscopic examination. However, the low-level and/or early stage infestations of *Psoroptes* generally have no visible scabs, making it impossible to collect skin scrapings for microscopic examination and leading to false negative diagnosis of *Psoroptes* infestations. In the artificial infestation experiment with *Psoroptes* mites, visible scabs were first observed until several weeks post-infestation, and this period can even extend up to several months in the field [4, 5]. In such situations, these cases without visible scabs cannot be identified by microscopy. Furthermore, it has been documented that even in skin scrapings, the detection rate of microscopy can be as low as 18% [6]. Therefore, microscopic detection in skin scrapings still faces challenges in terms of poor sensitivity for low-level and/or early stage mite infestations, but these animals misdiagnosed by microscopy could serve as potential sources of infestations in flocks. Consequently, seeking a sensitive diagnostic method for detecting all infested rabbits, including those with low-level and/or early stage infestations, is crucial for timely treatment of animals and reducing the risk of *P. ovis* mite transmission.

Understanding the pathogenesis of *Psoroptes* mite infestations is vital for providing direct clues on how to effectively control this parasite transmission in flocks. However, research progress on the pathogenesis of these mite infestations has made very limited advances in the past few decades. *Psoroptes* mite infestations can provoke a rapid profound cutaneous inflammation response within 24 h post-infection [7], and the serous exudate from this inflammatory response can provide sufficient food for the growth of the mite population [8]. Therefore, the use of anti-inflammatory drugs can reduce the mite burden and ameliorate the progression of *Psoroptes* infestations [9, 10]. Consequently, the identification of anti-inflammation molecules from *Psoroptes* might offer an alternative therapeutic approach for treating *Psoroptes* infestations in the future. Furthermore, *Psoroptes* mites secrete certain molecules to cope with intense inflammation and subsequent adaptive immunity for its long-time survival on the hosts. However, these anti-inflammation molecules of *Psoroptes* mites still remain unknown.

The proteins in cystatin family are known to be categorized into three major families comprising stefins (family I), cystatins (family II), and kininogens (family III) [11]. Cystatins belong to the family of cysteine protease inhibitors, and exist in all living organisms including mammals, plants, and parasites. In parasites, cystatins have been shown to exert dual functions by interacting with both parasite and host proteases. Therefore, these proteins not only participate in the regulation of essential physiological processes in parasites’ lifecycle, but also are involved in host immune modulation, such as inhibiting antigen processing and presentation, regulating pattern-recognition receptor signaling, and suppressing inflammatory responses [12, 13]. Members of the cystatins usually consist of ~ 120 amino acid residues with molecular masses of 13 ~ 15 kDa. They typically possess a conserved N-terminal glycine residue, a central QXVXG motif, and a C-terminal LW conservative site, all of which are important for the catalytic activity of cystatins [14]. Additionally, cystatins have been demonstrated as promising diagnostic antigens for various parasitic diseases, such as schistosomiasis [15], fascioliasis [16], and trichinellosis [17]. In *P. ovis*, the presence of cystatins has been confirmed through expressed sequence tag (EST) and transcriptome analysis [18, 19]. Beyond that, no further studies have been conducted on cystatin of *P. ovis.*

In this study, we identified a novel cystatin of *P. ovis* var. *cuniculi* from our transcriptomic data (data unpublished). We analyzed the transcriptional expression of cystatin (PsoCys) in mites, then cloned and prokaryotically expressed the cystatin of *P. ovis* var. *cuniculi* (rPsoCys), and evaluated the inhibitory activity of rPsoCys against proteases. Additionally, we investigated its serodiagnostic potential for detecting *P. ovis* var. *cuniculi* infestation in rabbits. Finally, we evaluated the anti-inflammatory capacity of rPsoCys in LPS-treated peripheral blood mononuclear cells (PBMCs).

## Methods

### Sequence analysis

The open reading frame (ORF) of PsoCys was predicted by ORF finder (http://www.ncbi.nlm. nih.gov/gorf/gorf.html). Its molecular weight and theoretical isoelectric point (pI) were predicted by ProtParam server (http://web.expasy.org/protparam/), and the signal peptide and transmembrane regions were predicted by signalP 5.0 (http://www.cbs.dtu.dk/Services/SignalP/) and TMHMM 2.0 (http://www.cbs.dtu.dk/services/TMHMM-2.0), respectively. Multiple sequence alignment of PsoCys and its orthologs was conducted by DNAMAN version 7.0 (Lynnon Biosoft, Quebec, Canada). The secondary and tertiary structures of PsoCys protein were predicted using Jpred 4.0 (http://www.compbio.dundee.ac.uk/jpred/) and SWISS-MODEL (https://swissmodel.expasy.org), respectively. The phylogenetic tree was constructed by neighbor-joining (NJ) method based on MEGA 11.0 (bootstrap = 1000).

### Transcriptional profiles of PsoCys at “fed” and “starved” mites of *P. ovis* var. *cuniculi*

New Zealand rabbits infested with *Psoroptes* mites were maintained in the Department of Parasitology, Sichuan Agricultural University (Sichuan, China), and these rabbits appeared to have typical symptoms of visible scab in the external ear canals. The “fed” and “starved” mites were collected essentially following the protocol as described by McNair et al. [20], and *Psoroptes* mites were identified under a microscope on the basis of the morphological characteristics described by Sweatman [21]. In brief, mixed-stage *P. ovis* var. *cuniculi* mites, including larvae, nymphs, and adults, were immediately isolated from ear scabs of the infested rabbits, and served as “fed” mite group. “Starved” mite group was also mixed-stage mites, but they were obtained by transferring the “fed” mites above onto a cardboard sprayed with sterilized saline and incubating them at 25 °C for 4 days under 80–90% relative humidity. The remaining four groups consisted of larvae, nymphs, females, and males of the “fed” mites, respectively. A single pool of mites from each group was used for transcriptional analysis, but each sample was tested in triplicate by quantitative real-time polymerase chain reaction (qRT-PCR).

Following the manufacturer’s instructions, total RNA was extracted from approximately 20 mg of mites in each group using the EASYspin Plus RNA kit (Aidlab Biotechnologies Co., Ltd., Beijing, China), and the RNA concentration and purity were estimated using a K5800 micro-spectrophotometer (Kaiao Technology Development Co., Beijing, China). The extracted RNA with an absorbance ration of OD_260_/OD_280_ between 1.8 and 2.0 was used for the subsequent cDNA synthesis. The cDNA synthesis was conducted in a 10 μL reaction volume using a commercial RT EasyTM II kit (ForeGene Biotech Co., Ltd., Chengdu, China), which contained 5 μL of 2 × RT OR-EasyTM Mix, 1 μL of total RNA (final concentration of 500 ng), and 4 μL of RNase-free ddH_2_O. The transcription profile of PsoCys gene was determined using TB Green® Permix Ex Taq™ Kit (TaKaRa, Dalian, China) by qRT-PCR (LightCycler® 96 System, Roche, Switzerland). The specific primers for PsoCys gene were 5′-TGTCACACGAAAAACTGGTGCT-3′ (forward) and 5′-GTCGTTCTAATTGTTCCAACGAACTC-3′ (reverse). The 20 μL reaction system consisted of 2 μL of the above cDNA template, 10 μL of TB Green *Premix Ex Taq* (2 ×), 0.8 μL of each primer (10 μM), and 6.4 μL of ddH_2_O. Amplification conditions were 95 °C for 5 min, 95 °C for 5 s, and 60 °C for 30 s, followed by 40 cycles at 95 °C for 5 s, 60 °C for 60 s, and 95 °C for 1 s. The transcriptional data were normalized to the mRNA level of the housekeeping β-actin gene using the 2^−ΔΔCt^ method [22]. For the “fed” mite group, the “starved” mite group was used as a control, and for “fed” mites at different developmental stages, the “larvae” mite group was used as a control.

### Cloning, expression, and purification of recombinant PsoCys protein

Live mixed-stage *P. ovis* var. *cuniculi* mites were obtained from the same infested rabbits as mentioned above. Total RNA extraction and cDNA synthesis were performed as mentioned above using EASYspin Plus RNA kit (Aidlab) and RT EasyTM II kit (ForeGene). The full-length coding sequence of PsoCys was amplified by Touchdown- polymerase chain reaction (PCR) using the following primers: 5′-ATGTTTCGATCAATTATTTT-3′ (forward) and 5′-TCAATTACTATCACATCTAAAATCAATCAATCT-3′ (reverse). Subsequently, the PsoCys without the signal peptide was amplified using the primers 5′-CGGGATCCAAATATTGTCACACGAAAAACT-3′ (forward) and 5′-CCCAAGCTTTCAATTACTATCACATCTAAAATCAATCAATCT-3′ (reverse), with the *BamH* I and *Hind* III sites underlined, respectively. PCR reaction was 25 μL consisting of 1.0 μL cDNA, 12.5 μL 2 × Taq PCR Master Mix, 1.0 μL each primer (0.05 μM), and 9.5 μL ddH_2_O. PCR amplification protocol was as follows: 94 °C for 5 min; 94 °C for 1 min, followed by 20 cycles of 65 °C for 45 s with a decrease of 0.5 °C each cycle; 72 °C for 45 s; 94 °C for 1 min, followed by 20 cycles of 55 °C for 45 s; and 72 °C for 45 s. The PCR products of PsoCys without the signal peptide were inserted into pET32a (+) vector (Invitrogen, Beijing, China) and transformed into *Escherichia coli* Rosetta (DE3), which was then induced by 1 mM isopropyl-β-D-thiogalactoside (IPTG) at 37 °C for 8 h. The soluble recombinant protein was harvested and dissolved in 8 M urea, followed by purification using a Bioscale TM Mini Nuvia TM IMAC Ni-Charged column (Bio-Rad, California, USA) with a step-wise imidazole concentration of 20, 50, and 100 mM imidazole. The purity of rPsoCys was determined by 10% sodium dodecyl sulfate–polyacrylamide gel electrophoresis (SDS-PAGE).

### Western blot analysis

The western blot analysis of rPsoCys was performed essentially according to our previous study [23]. Briefly, 0.833 μg of rPsoCys was separated by 10% SDS-PAGE. The *P. ovis* var. *cuniculi*-positive rabbit serum and negative rabbit serum were diluted 1:200 in PBS and membranes were incubated with horseradish peroxidase-labeled (HRP) goat-anti-rabbit IgG at a dilution of 1:3000 (Bioss, Beijing, China).

### Protease inhibitory assay

The protease inhibitory activity of rPsoCys was determined as previously described with some modifications [24]. Briefly, 0.72 μM human papain (Solarbio, G8430) and 0.72 μM bovine spleen cathepsin B (Sigma-Aldrich, C6286) were preactivated in the assay buffer (Papain: 340 mM sodium acetate, 60 mM acetic acid, 4 mM EDTA, 8 mM DTT, pH 5.5; Cathepsin B: 100 mM sodium acetate, 2 mM EDTA, 1 mM DTT, pH 5.5) for 5 min at room temperature, and then incubated with the increasing concentrations of rPsoCys (from 100 to 1200 ng) for another 5 min in the dark at room temperature. The substrate of a final concentration of 10 mM benzyloxycarbonyl (Z)-Arg-Arg-aminoethylcoumarine (AMC) (Sigma-Aldrich) was added to each well and the fluorescence intensity was measured by the Multiskan GO Spectrophotometer (Thermo Scientific, Vantaa, Finland) with excitation at 390 nm and emission at 440 nm. The equal volume of AMC (10 mM) and reaction buffer were used as the positive and negative controls, respectively. The 50% inhibition concentrations of enzyme activity (IC50) for rPsoCys was determined as previously described [25]. All reactions were performed in triplicate.

### Establishment of an indirect ELISA based on rPsoCys (rPsoCys-iELISA) and evaluation of its diagnostic potential for detecting *P. ovis* var. *cuniculi* infestations in rabbits

A total of 43 *P. ovis* var. *cuniculi*-positive sera were obtained from a farm in Chengdu city (confirmed by microscopic identification of mites in skin scrapings), and 45 *P. ovis* var. *cuniculi*-negative sera were collected from another farm with no history of *Psoroptes* infestations. Additionally, 48 rabbit sera were used for cross-reaction analysis, including *Eimeria stiedai*-positive sera (*n* = 20) and *Sarcoptes scabiei*-positive sera (*n* = 28), which were provided by the Department of Parasitology, Sichuan Agricultural University. These 136 rabbit sera were used to establish an indrect enzyme-linked immunosorbent assay based on rPsoCys(rPsoCys-iELISA) , and the protocol was performed essentially as previously described by Crowther [26]. In brief, the checkerboard titration test was carried out to optimize the working dilutions of rPsoC*ys* and serum. The 96-well plates were coated with purified rPsoCys by two-fold dilution in 50 mM carbonate buffer (pH 9.6) from 9.996 μg/well to 0.156 μg/well and incubated overnight at 4 °C. The plates were washed three times in phosphate buffered saline (PBS) incorporating 0.05% (v/v) Tween-20 (PBST, pH 7.4) and blocked with 5% (w/v) skim milk solution at 37 °C for 2 h. Following three washing cycles, 100 μL of *P. ovis* var. *cuniculi*-positive sera and *P. ovis* var. *cuniculi*-negative sera in a two-fold dilution (ranging from 1:25 to 1:800) were added to each well and incubated at 37 °C for 1 h. After three washes, the plates were incubated with 50 μL/well of HRP-labeled goat anti-rabbit IgG (1:3000 dilution, v/v) at 37 °C for 1 h. After further washes, 100 μL of 3,3′,5,5′-tetrmethylbenzidine solution (TMB) (TIANGEN, Beijing, China) were added to each well and incubated at 37 °C for 20 min. The reaction was stopped with 100 μL/well of 2 M H_2_SO_4_, and the optical densities (OD) at 450 nm were determined using a Multiskan GO Spectrophotometer (Thermo). The optimal working conditions were determined on the basis of the highest OD_450_ ration of positive serum to negative serum (P/N). The sensitivity and specificity of the rPsoCys-iELISA were calculated as described in our previous study [27]. Briefly, sensitivity was assessed using 43 *P. ovis* var. *cuniculi*-positive sera according to the formula: (ELSIA-positive sera/True positive sera) × 100%. Specificity was determined using 20 *Eimeria stiedai*-positive sera, 28 *Sarcoptes scabiei*-positive sera, and 45 *P. ovis* var. *cuniculi*-negative sera according to the formula: (ELSIA-negative sera/true negative sera) × 100%. The optimum cutoff value was determined when OD_450_ value had the highest sum of sensitivity and specificity using receiver operator characteristic curve (ROC) [28]. The area under the ROC curve (AUC) was calculated using MedCalc 19.0.7 [29]. Repeatability (intra-assay variability) was assessed by performing a single run with five *Psoroptes*-positive serum samples, while reproducibility (inter-assay variability) was determined by analyzing triplicate runs of five *Psoroptes*-positive serum samples [30]. The coefficients of variation (CV) were calculated as the follow equation: CV% = (SD/mean) × 100%.

To compare the diagnostic efficacy of rPsoCys-iELISA with traditional “gold standard” microscopy, we used both methods to test rabbit serum samples from artificial and field experiments. A total of 84 clinical rabbit sera and 65 artificially infested rabbit sera were subjected to rPsoCys-iELISA. The clinical serum samples were collected from a rabbit farm with a history of psoroptic mange, while the artificially infested sera were collected from infested rabbits (*n* = 50) and non-infested rabbits (*n* = 15), respectively. The artificial infestations of rabbits with *Psoroptes* and the collection of sera were performed according to the protocol described previously [5]. Additionally, skin scraping samples were collected in presence of scabs on the external auditory canal from the clinically and experimentally infested rabbits, and were further examined by microscopic [31].

### Stimulation of rabbit PBMCs and transcriptional expression of Toll-like receptor signaling pathway-related molecules

The isolation of rabbit PBMCs was performed according to the procotol described in our previous study [23]. PBMCs from the New Zealand White rabbit were isolated by Ficoll lymphocyte separation solution (Beijing Solai Bao Technology Co., Led., Beijing, China) and resuspended in RPMI-1640 medium (Hyclone, South Logan, USA) containing 10% fetal bovine serum (PAN-Biotech GmbH), 100 U/mL of penicillin (Beijing Solai Bao Technology Co., Led., Beijing, China), 0.1 mg/mL of streptomycin (Biotopped, China), and 0.0025 mg/mL of amphotericin B (Biotopped, China). PBMCs (1 × 10^6^ cells/mL) were seeded into a 96 well-plate (TPP, Trasadingen, Switzerland) and incubated for 24 h at 37 °C in 5% CO_2_.

To determine the optimum stimulation conditions, PBMCs were treated with different concentrations of rPsoCys (the final concentrations of 0.05, 0.5, 1, 2, 4, 8, 10, and 20 μg/mL) and different incubation times (1, 2, 4, 8, and 12 h). Simultaneously, 1 μg/mL inactive-rPsoCys (heat-denatured treatment), 1 μg/mL pET32a protein, 0.03 μg/mL lipopolysaccharide (LPS), and 0.01 M PBS (pH 7.4) were used as control groups, respectively. Each treatment was conducted in triplicate. After incubation at 37 °C with 5% CO_2_ for 12 h, the CCK-8 kit (Beyotime Biotech, Haimen, China) was used to detect cell activity. The OD_450_ value of each well was measured using a Multiskan GO Spectrophotometer (Thermo).

The PBMCs were seeded into a 96 well-plate at a concentration of 1 × 10^6^ cells/well and incubated at 37 °C with 5% CO_2_ for 24 h. The cells were treated with LPS at a final concentration of 0.03 μg/mL at 37 °C with 5% CO_2_ for 4 h, followed by stimulation with the optimal incubation concentration of rPsoCys and incubation time. Simultaneously, 1 μg/mL inactive-rPsoCys, 1 μg/mL pET32a protein, and 0.01 M PBS (pH 7.4) were used as control groups. Each treatment was conducted in triplicate. After harvesting the cells, RNA extraction and cDNA synthesis were carried out using EASYspin Plus RNA kit (Aidlab) and RT EasyTM II kit (ForeGene) as previously described in this study. The transcription levels of the toll-like receptor signaling pathway-related molecules were detected by qRT-PCR with the β-actin gene as an endogenous control. The specific primers for target gene amplification in PBMCs were presented in Table 1. For qRT-PCR, the TB Green Permix Ex Taq^TM^ Kit (TaKaRa was used, and the amplifications were conducted on the ABI7500 FAST real-time PCR System (Applied Biosystems, Forster, USA). The reaction volume was 20 μL, containing 2 μL of cDNA template, 10 μL of TB Green *Premix Ex Taq* (2 ×), 0.8 μL of 10 μM each primer and 6.4 μL of ddH_2_O. The amplification procedures were as follows: 95 °C for 5 min, followed by 40 cycles at 95 °C for 5 s, 60 °C for 30 s, and an additional melting curve step at 60–95 °C. Relative mRNA expression level of the targeted genes was calculated using the 2^−△△Ct^ method [22].

**Table 1:** The primers for transcriptional analysis of TLR pathway

Genes	Primers (5′–3′)	Size (bp)
*β*-actin	F: TGAATTGCCTGATGGTCAAG	167
	R: TGGCGAACAAGTCTTTACGG	
TLR 1	F: GTCCAGAGTGAGTGGTGCCATTATG	166
	R: CCAAGTAAGTCCTCCGTGCCATG	
TLR 2	F: CTGTCTGTCACCGAACCGAATCC	122
	R: AAGTCCAGTAACTCAGGCACATAAGC	
TLR 3	F: GAGTGATGCGTTCTCCTGGTTGG	90
	R: CTCCATTCCTGTCCTGTGAGTTCTTG	
TLR 4	F: GAGGAGGCTGTTGGTGGAAGTTG	128
	R: GCACACTGAATACTGACACGCTAATG	
TLR 5	F: GGATGCCGTCTTGCGAGATGG	82
	R:GCTGTGGATCTTATTGAAGGAGAGGTC	
TLR 6	F: AGGCACCAAGCACATTCCAGTTC	168
	R: CCAGAGACGGCATATCCTTAGTCATG	
TLR 8	F: GCATACGGCGGAGAAGAAGAC	138
	R: CGAGCAGCACGAAGACGATGAC	
TLR 9	F: CATCACGGACACCACAGCCTTC R: CTGAGCGAGCGGAAGAAGATGC	171
TLR 10	F: TCCGACTCGTACCTCTGTGAATACC	199
	R: TCCATTGACCTAGCATCCTGAGATACC	
TLR 11	F: ACACTGGAGGAGGTACTGAAGGAAG	121
	R: GCAACAGCAGCCGAAGGAGAC	
TLR 12	F: AGGTCTTGGTGCTGATGGAGGAG	179
	R: CGCTCTGATGTGCCAGTAGGTTC	
NF-κB	F: CGGAGGCTGAGAGGCGAGAG	171
	R: CAGGAGACTTGCTGTCGTGGATG	
p38	F: TTCAGTCTTTGACCCAGATGC	178
	R: GGGCTGCTGTGATTCTCTTATC	
MyD88	F: ATCGAGGAGGACTGCCAGAAGTAC	169
	R: TCCGAGACGACCACCACCATC	
IL-8	F: CTCTTGGCAACCTTCCTGCTCTC	83
	R: CACTGGCATCGAAGCTCTGTACC	
IL-10	F: ATGCCGCAAGCTGAGAACCAC	147
	R: CTTCACCTGCTCCACTGCCTTG	
IFN-γ	F: TTTCCCAAGGATAGCAGTGGT	146
	R: TGAAGCCAGAAGTCCTCAAAA	

### Statistical analyses

All data were presented as mean ± SD. The significant difference between groups was tested by one-way analysis of variance (ANOVA) with Mann–Whitney *U* test (SPSS statistics 23.0). A *P*-value < 0.05 was set as statistically significant.

## Results

### Sequence analyses of PsoCys

**Fig. 1:**
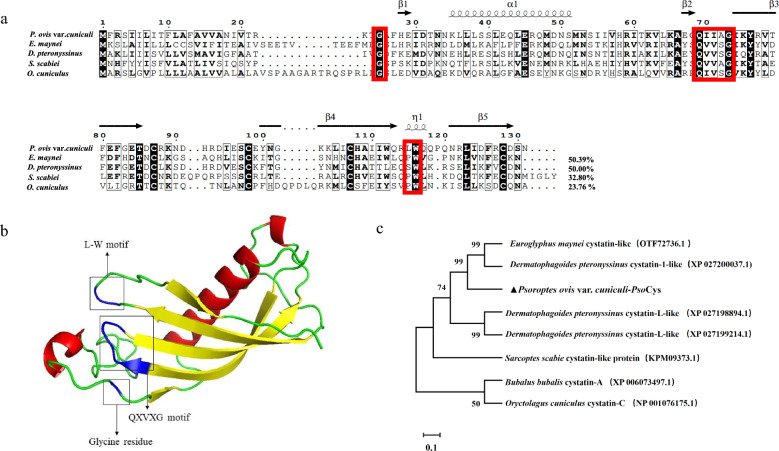
Multiple sequence alignment, predicted tertiary structures, and phylogenetic analysis. **a** Multiple alignment of amino acid residues deduced by PsoCys with homologous sequences of related proteins of other parasites, such as *Sarcoptes scabiei* (GenBank: KPM09373), *Dermatophagoides pteronyssinus* (Genbank: XP027198894.1), *Euroglyphus maynei* (GenBank: OTF72736), and *Oryctolagus cuniculus* (GenBank: BAA75921.1). (Red frame: conserved domains in cystatins family). **b** Tertiary structure of *PsoCys*. Schematic representation of *Psoroptes ovis* var. *cuniculi* with conserved residues (C-terminal LW motif, a central QXVXG motif, N-terminal glycine residue). **c** Phylogenetic analysis of cystatin PsoCys was conducted by constructing a phylogenetic tree using the neighbor-joining (NJ) method in the MEGA 11 program on the basis of the alignment of amino acid sequences from various species. The numbers at nodes are the bootstrapping frequency (Bf) values of 1000 replications

The PsoCys-open reading frame was 396 bp (GenBank number: PP735377) and encodes a protein of 131 amino acids with a predicted molecular mass of 15.3 kDa and a theoretical pI of 5.35. PsoCys was predicted to possess a signal peptide residue 1–17 but lacks transmembrane region. On the basis of the predicted secondary structure, PsoCys possessed three highly conserved type 2 cystatins residues, including a N-terminal glycine residue, a central QXVXG motif, and a C-terminal LW motif (Fig. 1a, b), suggesting that it belongs to the type II cystatin superfamily (family II) [32, 33]. The amino acid sequence of PsoCys showed 32.8–50.4% identity with cystatins from three other mites, including *Euroglyphus maynei*, *Dermatophagoides pteronyssinus*, and *Sarcoptes scabiei* (Fig. 1a). The NJ tree based on the amino acid alignment of cystain indicated that *P. ovis* presented a closer relationship to *E. maynei* and *D. pteronyssinus* than to its hosts (rabbit and buffalo) (Fig. 1c).

### Transcriptional profiles of PsoCys in *P. ovis* var. *cuniculi*

**Fig. 2:**
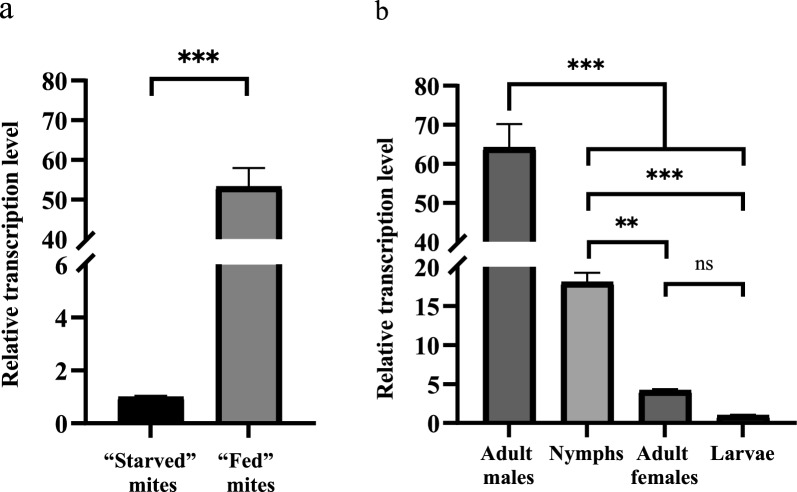
Relative transcriptional profiles of PsoCys in “starved” and “fed” mites (**a**) and different development stages in “fed” mites (**b**). The internal reference gene was β-actin in the study. Data are represented as the mean with standard deviation (SD) in triplicate. ***: the difference between samples is statistically significant (*P*<0.001), **: the difference between samples is statistically significant (*P* < 0.01), ns: no significant difference between samples

In the mixed stage of mites, the PsoCys transcription level in “fed” mite group was significantly higher than that in “starved” mite group (*P* < 0.001) (Fig. 2a). Among the different lifecycle stages of “fed” mites, PsoCys showed differential expression across the selected lifecycle stages, and adult males had the highest transcription level compared with larvae, nymphs, and adult females (*P* < 0.001) (Fig. 2b). Additionally, there was a significant difference between nymphs and larvae (*P* < 0.001), as well as between nymphs and adult females (*P* < 0.01), while no significant difference was observed between adult females and larvae (*P* > 0.05) (Fig. 2b).

### Expression and western blotting of rPsoCys

**Fig. 3:**
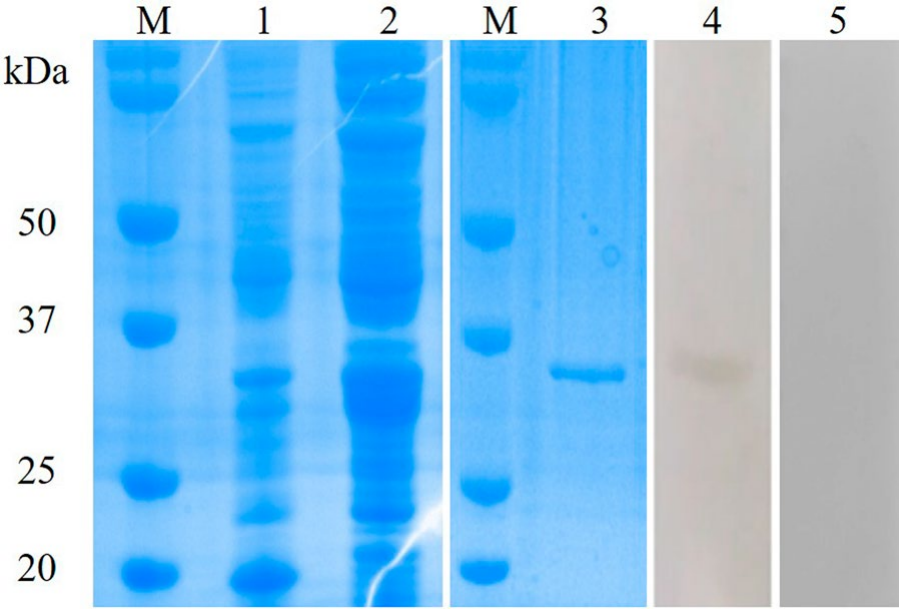
Expression and western blotting of recombinant PsoCys. Lane M: protein molecular weight marker. Lane 1: pET32a (+)-vector protein induced by IPTG. Lane 2: pET32a (+)-PsoCys induced by IPTG. Lane 3: the purified rPsoCys. Lane 4: the purified rPsoCys immunoblotted with *Psoroptes ovis* var. *cuniculi*-positive sera. Lane 5: the purified rPsoCys immunoblotted with the negative serum

The full-length PsoCys cDNA without the signal peptide was amplified, and then subcloned into pET-32a (+) vector for recombinant protein expression. The rPsoCys were successfully obtained in soluble form with the expected molecular weight of ~ 35 kDa (including a ~ 20 kDa epitope tag fusion peptide) (Fig. 3). Western blotting showed that purified rPsoCys could react with *P. ovis* var. *cuniculi*-positive serum but did not react with negative serum.

### Proteinase inhibition assay

**Fig. 4:**
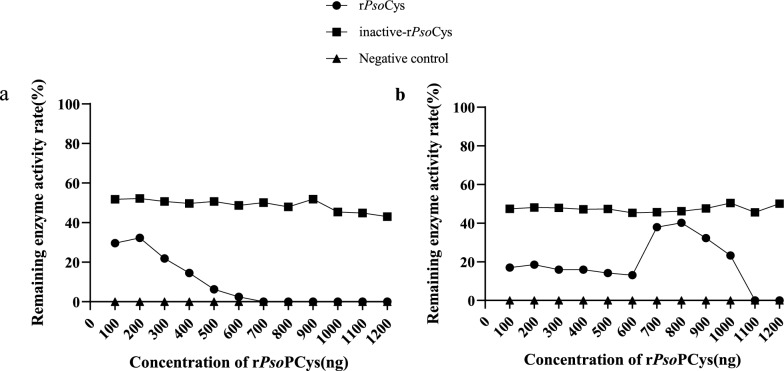
Inhibition assays with rPsoCys against papain (**a**) and cathepsin B (**b**). The abscissa indicates the concentration of rPsoCys. The ordinate indicates residual activity of papain and cathepsin B (in percentage)

The rPsoCys exhibited potential inhibitory activity against papain and cathepsin B. Specifically, the inhibitory activity of rPsoCys against papain was stronger than that against cathepsin B, resulting in a lower IC_50_ value for papain compared with cathepsin B (papain: 51.64 ng; cathepsin B: 128.6 ng) (Fig. 4a, b). It was noteworthy that papain was inhibited in a dose-dependent manner, while cathepsin B was not.

### Establishment rPsoCys-iELISA and its evaluation of detecting *P. ovis* var. *cuniculi* infestations in rabbits

**Fig. 5:**
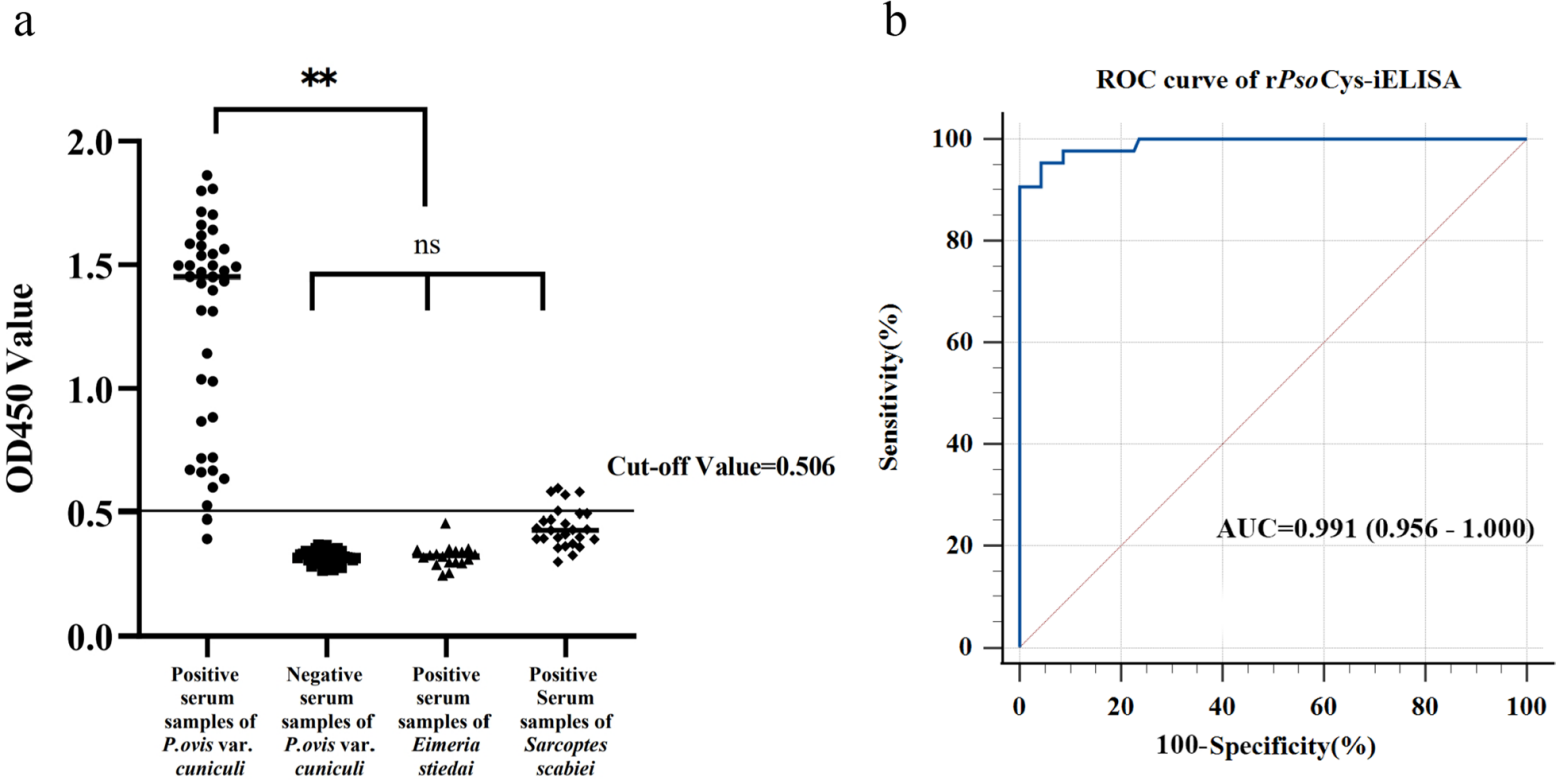
Evaluation of rPsoCys-iELISA. **a** The sensitivities and specificities of rPsoCys-iELISA. The ordinate: OD_450 nm_ value of the serum samples. The fine horizontal line: the critical value of positive detection (cutoff value). The abscissa: different kinds of serum samples. **b** ROC analysis of rPsoCys-iELISA. **: the difference between samples is statistically significant (*P* < 0.01), ns: no significant difference between samples

The optimal working conditions for rPsoCys-iELISA were determined as the antigen coating concentration of 8.33 μg/mL rPsoCys and the primary serum dilution of 1:200. By analyzing the ROC curve, the optimal cutoff value for rPsoCys-iELISA was determined as an OD_450_ value of 0.506. The sensitivity and specificity of rPsoCys-iELISA was 95.4% (41/43) and 95.7% (89/93), respectively (Fig. 5a). The AUC for rPsoCys-iELISA was 0.991, indicating an excellent ability to discriminate between test-positive and test-negative results (Fig. 5b). The intra- and inter-assay of CVs for rPsoCys-iELISA were 5.0% and 4.5%, respectively, suggesting that this test was stable and reproducible.

**Fig. 6:**
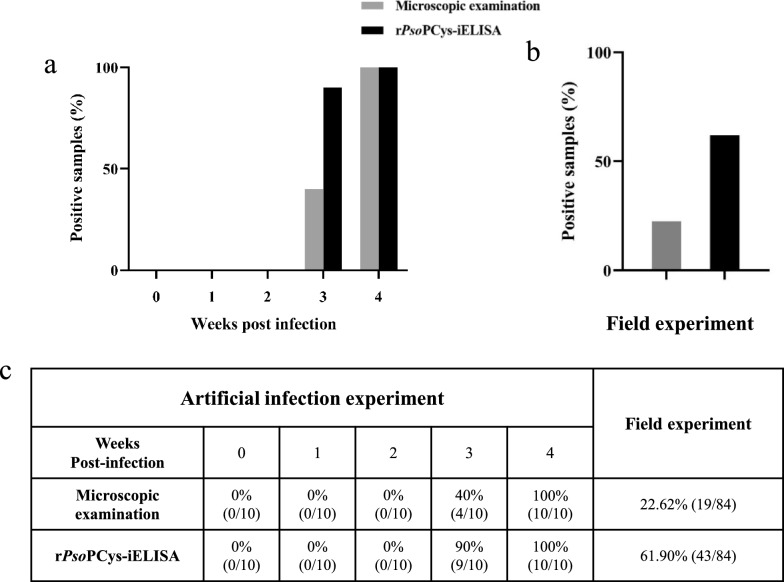
Comparison of positive rate detected by microscopic examination and rPsoCys-iELISA in artificial infestation experiment and field experiment. **a** Artificial infestation experiment. **b** Field experiment. **c** Summary of the positive rate for detecting *Psoroptes* mite infestations using two tests

Since microscopic examination requires collecting scabs from cases for mite identification, it is essential to observe the presence of scabs in cases. In artificial infestation experiment, all rabbits had no visible crust at 1 and 2 wpi, making it impossible to collect skin scrapings for microscopic examination and thus judged as *Psoroptes*-negative. At 3 wpi, only four out of ten rabbits had visible scabs, and microscopic examination of these skin scrapings identified the presence of *Psoroptes* mites, resulting in a 40% positivity rate. At 4 wpi, all rabbits (*n* = 10) had visible scabs and microscopic examination yielded a 100% positive rate (Fig. 6a). For rPsoCys-iELISA, no rabbit tested positive at 1 wpi and 2 wpi. At 3 wpi, nine rabbits tested positive with a 90% (9/10) of detection rate, and this further increased to 100% at 4 wpi (Fig. 6a). It is worth noticing that, at 3 wpi, rPsoCys-iELISA was able to detect five more positive samples among the six microscopy-negative samples, suggesting that this ELISA can detect low-level and/or early stage mite infestations (Fig. 6c).

In field investigation, 43 rabbits tested positive in rPsoCys-iELISA, resulting in a positive rate of 61.9% (43/84) (Fig. 6b). Only 19 out of 84 rabbits had visible scabs in the external auditory canal, and further microscopic examinations revealed the presence of *Psoroptes* mites, yielding a positive rate of 22.6% (19/84) (Fig. 6c). The detection rate of rPsoCys-iELISA was 2.74 times higher than that of microscopic examination, indicating that this test was more sensitive than microscopic examination.

### rPsoCys suppressed transcriptional expressions of Toll-like receptor signaling pathway-related molecules in LPS-stimulated rabbit PBMCs

**Fig. 7:**
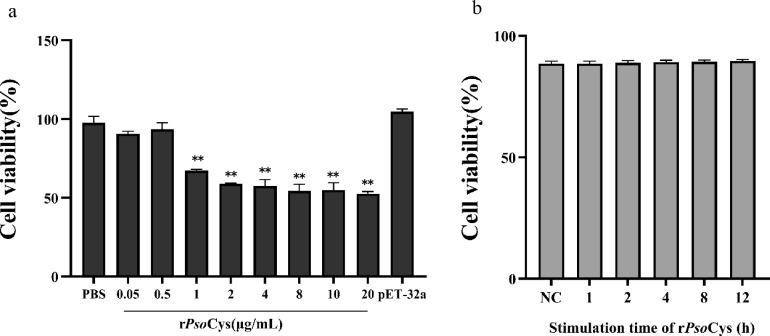
Effects of different concentrations (**a**) and incubation time (**b**) of rPsoCys on the cell viability of PBMCs. PBS, PBS group (negative control); rPsoCys, recombinant *Psoroptes ovis* var. *cuniculi* cystatin; pET-32a, pET32a (+) vector control. ** represents the difference between samples is statistically significant (*P* < 0.0), No annotation represents no significant difference between samples

To analyze the effect of rPsoCys on cell viability and determine the optimal concentration and time of rPsoCys in PBMCs, we carried out cell proliferation tests by CCK-8 method. The results showed that 0.5 μg/mL rPsoCys had no effect on cell viability, but 1–20 μg/mL rPsoCys significantly reduced cell viability (*P* < 0.01) (Fig. 7a). Therefore, for excluding the effect of cell viability in the following experiments, we selected 0.5 μg/mL rPsoCys as the optimal concentration for incubation with PBMCs. Subsequently, the optimal concentration of rPsoCys was incubated with rabbit PBMCs for 1, 2, 4, 8, and 12 h, and the results showed that there was no significant difference in cell viability between rPsoCys group and PBS group (Fig. 7b). Finally, we selected 4 h as the optimal incubation time in the following tests.

**Fig. 8:**
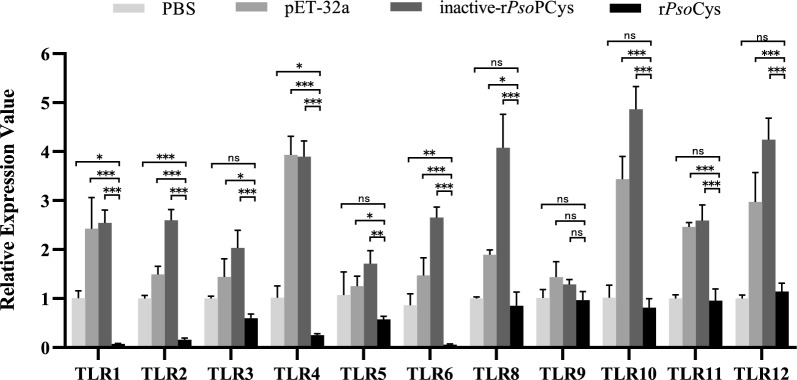
Relative mRNA expression patterns of TLRs in rabbit PBMCs treatment with 0.5 μg/mL rPsoCys for 4 h. PBS, PBS group (negative control); LPS, positive control with lipopolysaccharide; rPsoCys, recombinant *Psoroptes ovis* var. *cuniculi* cystatin; inactive-rPsoCys, recombinant *Psoroptes ovis* var. *cuniculi* cystatin by heat-denatured treatment; pET-32a, pET32a (+) vector control. *** represents the difference between samples is statistically significant (*P*<0.001),  ** represents the difference between samples is statistically significant (*P* < 0.01), * represents the difference between samples is statistically significant (*P* < 0.05), ns: no significant difference between samples

**Fig. 9:**
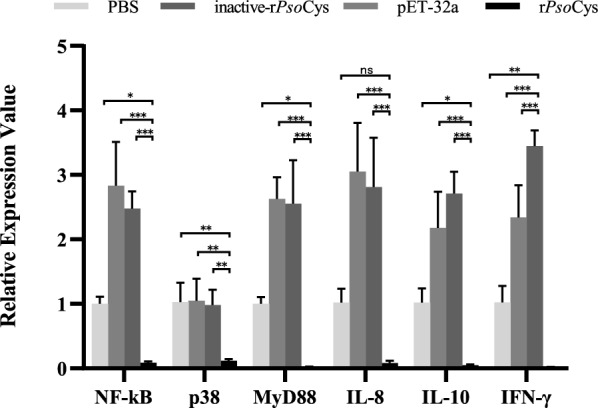
Relative expression of key molecules in Toll signaling pathway of PBMCs treated with rPsoCys. PBS, PBS group (negative control); rPsoCys, recombinant *Psoroptes ovis* var. *cuniculi* cystatin; inactive-rPsoCys, recombinant *Psoroptes ovis* var. *cuniculi* cystatin by heat-denatured treatment; pET-32a, pET32a (+) vector control. *** represents the difference between samples is statistically significant (*P*<0.001), ** represents the difference between samples is statistically significant (*P* < 0.01), * represents the difference between samples is statistically significant (*P* < 0.05), ns: no significant difference between samples

To evaluate antiinflammatory capacity of PsoCys, LPS-stimulated rabbit PBMCs were treated with rPsoCys, and the transcriptional expressions of Toll-like receptor signaling pathway-related molecules in PBMCs were investigated by qRT-PCR. As shown in Figs. 8 and 9, rPsoCys significantly downregulated the mRNA expressions of TLRs (TLR1, 2, 4, 6) and their down-stream molecules (NF-κB, p38, MyD88, IL-10 and IFN-γ) in LPS-stimulated PBMCs. Additionally, compared with pET-32a group and inactive-rPsoCys group, there were significant differences in all targeted genes (except for TLR9) in rPsoCys group (Figs. 8, 9). These results suggested that rPsoCys could suppress the inflammatory responses in LPS-stimulated rabbit PBMCs.

## Discussion

Cystatins are known as a family of cysteine protease inhibitors and involved in development of parasite and modulation of host immune response, including inhibition of antigen processing and presentation, regulation of pattern recognition receptor signaling, and suppression of inflammatory responses [12, 13]. Although cystatins are likely important, their biological functions in *P. ovis* are still poorly understood. Herein, we identified and functionally characterized a novel cystatin of *P. ovis* var. *cuniculi* for the first time. Our results revealed that PsoCys was a functional cysteine protease inhibitor of type II cystatins, and served as a potential serodiagnosis antigen for low-level and/or early-stage infestations of *P. ovis*, as well as appeared to be an anti-inflammatory property by suppressing Toll-like receptor signaling pathway-related molecules.

In this study, PsoCys showed limited sequence identity (32.8–50.4%) with the orthologous cystatins from other three mite species, but it exhibited structural conservation with type II cystatins, containing N-terminal glycine residue, a central QXVXG motif, and an L–W pair at the C-terminus [34], and these three conservation structural regions are closely related to the protease inhibitory activity of cystatins. The QXVXG and P–W or L–W motifs are respectively folded into a hairpin loop, and these two loops formed a tripartite wedge that interacts with the active site groove of the proteinase, providing the binding energy to stabilize this interaction [35]. The N-terminal glycine, together with these two loops, forms a hydrophobic wedge-shaped edge that is highly complementary to the active site of the cysteine proteinases, thereby inhibiting protease activity [36]. In the present study, PsoCys possessed the above three conserved regions (Fig. 1), suggesting that it might have protease inhibitor activity. This speculation was confirmed by the proteinase inhibitory assays, which showed that rPsoCys had inhibitor activity against papain and cathepsin B (Fig. 4). In the mixed-development stages of mites, the transcriptional level of cystatin was higher in “fed” mites than in “starved” mites, and this gene was expressed across all selected developmental stages in “fed” mites (Fig. 2). Cystatin has been proven to play an important role in the successful feeding process of ticks  on hosts [37]. Therefore, we speculated that *Psoroptes* mites might secrete cystatin to interact with parasite and host proteases, regulating their own digestion process and modulating host’s immune response, thereby facilitating their successful feeding on the hosts.

Microscopy, as the current “gold standard” for diagnosis of *P. ovis* infestations, has disadvantages of being inefficient, time-consuming, and a heavy workload. Furthermore, this method had low diagnostic sensitivity in identifying true-positive status in individuals with low mite burden infestations and/or early stage mite infestations, and the detection rate of microscopy has been reported to be as low as 18% [6, 38]. Therefore, the identification of all *P. ovis* infestation cases in flocks is critical for timely treatment and preventing disease transmission. Hosts exposed to *P. ovis* can produce host-specific antibodies [4, 39], and this specific antibody can be detected by serological method in the early stage of *P. ovis* infestations, although there are no visible scabs on the hosts at this stage [5]. Consequently, serological diagnosis has rapidly developed as one of the alternative methods to microscopy examination for detecting *P. ovis* infestations, including Pso o2-iELISA [28], PsoTrc-iELISA [40], PsoAK-iELISA [5], PsoSP2-iELISA, and Pso c27-iELISA [27]. Previous studies have confirmed that cystatins were considered promising diagnostic antigens for paragonimiasis [15], fascioliasis [16], and trichinellosis [17]. Therefore, in this study, we established iELISA method on the basis of rPsoCys and evaluated its serodiagnostic potential for detecting *P. ovis* var. *cuniculi* infestation in rabbits. Our results showed that rPsoCys-iELISA exhibited excellent diagnostic accuracy for detecting *P. ovis* infestation in rabbits (AUC = 0.991, specificity = 95.7%, sensitivity = 95.4%, CV = 5.0% and 4.5%). Upon further artificial and field experiments, it was found that rPsoCys-iELISA was more sensitive than microscopy, especially in detecting low-level and/or early stage mite infestations. At 3 wpi, rPsoCys-iELISA detected serum positivity in five out of six rabbits without visible scabs, while these rabbits were judged as *Psoroptes*-negative by microscopic examination. This conclusion was also confirmed in field investigation, showing that rPsoCys-iELISA could detect 24 more positive samples among 65 microscopy-negative samples. These results suggested that rPsoCys-iELISA had advantages over microscopy for detecting low-level and/or early stage infestations in rabbits. Surprisingly, two rabbits were confirmed as *Psoroptes*-positive by traditional microscopic examination, however, these two serum samples were not detected as seropositive by rPsoCys-iELISA. This may be related to the fact that these two rabbits have a lower immune reactivity to *Psoroptes* mite infestations, resulting in a lower antibody titer. Moreover, rPsoCys-iELISA showed non-specific cross-reaction with four rabbit sera from *Sarcoptes scabiei* (4/28), and this serologic cross-reactions between *P. ovis* and *S. scabiei* infestations had been demonstrated in other studies [5, 41, 42]. Fortunately, the ecto-parasitic diseases caused by *Psoroptes* and *Sarcoptes* mites can be effectively treated with the same chemical acaricidal drugs, such as ivermectin, doramection, and moxidection [43, 44]. To sum up, rPsoCys-iELISA was a potential serodiagnosis test for detecting low-level and/or early stage infestations with *P. ovis* var. *cuniculi*.

Many immunological processes rely on extracellular and intracellular activities of proteases and their respective inhibitors. Major histocompatibility complex (MHC) class II surface molecules of antigen-presenting cells (APCs) present antigenic peptides to CD4^+^ T cells [45], and cathepsin B is involved in the production of full antigenic peptides and their loading onto MHC class II molecules [46]. Thus, inhibiting the proteolytic activity of cathepsin B could lead to a reduction of MHC class-II-mediated antigen presentation by APCs. Cystatins can bind and obstruct the active site of proteases to inhibit lysosomal protease activity, suggesting its role in the regulation antigen processing and presentation of APCs, and this function of parasite cystatins has been confirmed in *Nippostrongylus brasiliensis* [47], *Onchocerca volvulus* [48], and *Brugia malayi* [49]. In our study, rPsoCys displayed a strong inhibitory activity against cathepsin B, thus we speculate that *Psoroptes* mites might employ cystatins to affect the processing and presentation of antigens by inhibiting cathepsin B activity to regulate host immune response, thus facilitating the survival of *Psoroptes* mites. Papain can induce degradation of tight junction proteins in skin and compromise the integrity of the skin barrier, subsequently increasing vascular leakage and inciting the inflammatory response [50]. It also induces notable superoxide production and degranulation of eosinophils [51], resulting in severe allergic reactions that are toxic to both parasite and host tissue. In the present study, rPsoCys had been confirmed, efficiently inhibiting papain activity, suggesting *Psoroptes* mites might utilize cystatin to inhibit host papain activity, thereby reducing excessive inflammatory response and allergic reaction. Thus, this alleviating of the inflammation response could be beneficial to mite survival on the host’s skin. However, the anti-inflammation effect of PsoCys cannot be too strong, because the exudations from inflammatory response served as a food source of *Psoroptes* mites [8]. Therefore, it is essential for *Psoroptes* to simultaneously secret both pro- and anti-inflammatory molecules to maintain a delicate balance between pro- and anti-inflammatory response within the skin microclimate. This balance is beneficial to both host and mites, thereby achieving persistent infestations of *Psoroptes* mites. Interestingly, parasite cystatins can regulate inflammatory response via Toll-like receptor (TLR) signaling, which effects the production of proinflammatory cytokines such as IL-8, IL-10, and IFN-γ, ultimately regulating local or systemic immune responses [52–54]. Studies demonstrated that cystatin of *Dermacentor silvarum* and *Leishmania donovani* inhibited TLR signaling transduction of TLR 2, 4 and the activation of NF-κB to regulate the inflammation response [55]. Our study observed the similar results that rPsoCys suppressed LPS-induced activation of the Toll-like receptors signal pathway with the decreased gene expressions including TLR1, 2, 4, and 6, and NF-κB, p38, and MyD88 in rabbit PBMCs. Ultimately, the inhibition of TLR-induced signal transduction by rPsoCys resulted in the reducing gene expression of downstream proinflammatory cytokines of IL-10 and IFN-γ in LPS-stimulated PBMCs. This similar function of cystatins in downregulating of the cytokine production has been confirmed in *Trichinella spiralis* [24] and *Schistosoma japonicum* [56].

## Conclusions

PsoCys belonged to the type II cystatin with the typical conservative structural regions, and served as a potential serological diagnostic antigen for detecting low-level and/or early stage infestations of *P. ovis* var. *cuniculi*. It was also a novel anti-inflammatory molecule of *Psoroptes* mites.

## Data Availability

The nucleotide sequence of cystatin gene from *P. ovis* var. *cuniculi* in this article is available in the GenBank database under accession no. PP735377. The other data supporting our findings and conclusions are available in the article.

## Abbreviations

*P. ovis*: *Psoroptes ovis*
*P. ovis* var. *cuniculi*: *Psoroptes ovis* var. *cuniculi*
PsoCys: Cystatin of *P. ovis* var. *cuniculi*
PBMCs: Peripheral blood mononuclear cells
ORF: Open reading frame
SDS-PAGE: Sodium dodecyl sulfate–polyacrylamide gel electrophoresis
AMC: Benzyloxycarbonyl (Z)-Arg-Arg-aminoethylcoumarine
PBS: Phosphate-buffered saline
TMB: 3,3′,5,5′-Tetrmethylbenzidine solution
ROC: Receiver operator characteristic
CV: Coefficients of variation
SD: Standard deviation
PCR: Polymerase chain reaction
qRT-PCR: Quantitative real-time polymerase chain reaction
pI: Isoelectric point
NJ: Neighbor-joining
Bf: Bootstrapping frequency
IPTG: Isopropyl-β-d-thiogalactoside
HRP: Horseradish peroxidase
OD: Optical densities
PBS: Phosphate-buffered saline
